# Retrovirus-Induced Immunosuppression: Role of the Transmembrane Envelope Protein

**DOI:** 10.3390/v18070740

**Published:** 2026-07-03

**Authors:** Joachim Denner

**Affiliations:** Institute of Virology, Free University Berlin, 14163 Berlin, Germany; Joachim.Denner@fu-berlin.de; Tel.: +49-175-591-7006

**Keywords:** retroviruses, transmembrane envelope protein, immunosuppressive domain, immunosuppression

## Abstract

Retroviruses induce immunosuppression in their infected hosts. This phenomenon is well described for the immunodeficiency viruses, with human immunodeficiency virus type 1 (HIV-1) representing the best-studied example, but it also occurs in other retroviral infections. Immunosuppressive properties were first characterized in murine leukemia viruses (MuLV). Additional well-studied examples include feline leukemia virus (FeLV) and koala retrovirus (KoRV). Investigations into the mechanisms underlying retrovirus-induced immunosuppression revealed that not only inactivated viral particles but also their purified transmembrane (TM) envelope proteins exhibit immunosuppressive activity. However, in certain retroviral infections, additional viral proteins contribute to the immunosuppression in vivo. Within the TM envelope proteins, a highly conserved region—designated the immunosuppressive (isu) domain—was identified. Synthetic peptides corresponding to this domain suppress a wide range of in vitro immune responses, possibly by regulating Ras-Raf-MEK-MAPK and PI3K-AKT-mTOR pathways. They modulate cytokine release and alter gene expression in immune cells, mirroring the activity of the corresponding TM envelope protein. Mutations in the sequence abrogate the effect. Numerous TM envelope proteins have demonstrated immunosuppressive activity in vivo in a tumor rejection model, and mutations within the isu domain also abrogate this function. These studies have important implications for reproduction, particularly through the immunosuppressive syncytins in the placenta, for tumor development, where similar mechanisms may protect cancer cells from the host immune system, and for vaccine development and xenotransplantation. Notably, immunization with TM envelope proteins carrying mutations in the isu domain elicits stronger immune responses compared with the wild-type proteins. Finally, the potential of retroviral TM envelope proteins to protect xenotransplants from immune rejection will be discussed.

## 1. Introduction

Many viruses induce immunosuppression in the infected host to facilitate successful infection. One of the most prominent examples is infection with the human immunodeficiency virus (HIV), which leads to acquired immunodeficiency syndromes (AIDS) [1]. Immunosuppression was observed with different classes of viruses in different species [1,2,3,4,5,6].

Virus-induced immunosuppression can occur through several mechanisms, including infection of immune cells, interaction of viral proteins with components of the immune system, or the induction of T cell-mediated immunopathology targeting infected immune cells [2,7]. The measles virus represents a well-studied example in which viral proteins actively suppress T-cell function by interfering with signaling pathways essential for T-cell activation [3].

## 2. Retrovirus Infections Induce Immunosuppression

Infection with retroviruses induces immunosuppression in the infected hosts. This is true for HIV [1] and other immunodeficiency viruses, such as the simian immunodeficiency viruses (SIVs) [8], the feline immunodeficiency virus (FIV) [9], and other lentiviruses, including equine infectious anemia virus (EIAV) in horses [10] and caprine arthritis encephalitis virus (CAEV)/Maedi-Visna virus in goats and sheep [11]. Infections with these viruses cause persistent infections with progressive immune dysfunction, which are usually associated with different opportunistic infections including pneumocystis pneumonia, tuberculosis, and candidiasis.

It is important to note that SIVs in their natural hosts do not cause immunosuppression. However, following trans-species transmission to humans or macaques, these viruses can lead to AIDS. This contrast suggests that natural hosts have evolved mechanisms of resistance, enabling them to sustain high viral loads without progressing to disease [12]. Elucidating the evolutionary adaptations underlying this resistance in natural SIV hosts may offer valuable insights for the development of effective therapeutic strategies against HIV infection in humans [13].

Sooty mangabeys are Old World monkeys that carry high levels of SIV in their blood yet do not develop signs of disease. Comparative genomic analysis between sooty mangabeys and AIDS-susceptible primates identified 34 immune-related genes with notable sequence diversity [14]. Among these, intercellular adhesion molecule 2 (ICAM-2) and toll-like-receptor 4 (TLR4) exhibited the greatest amino acid variation relative to macaques. In sooty mangabeys, ICAM-2 is nonfunctional, while TLR4 contains an additional 17 amino acids at the C-terminus. This C-terminal extension is also present in other AIDS-resistant primates and has been associated with reduced inflammatory responses, which may contribute to disease resistance in these species.

Retrovirus-induced immunosuppression was first reported in 1960 in mice infected with murine leukemia virus (MuLV) [15]. Building on these early findings, multiple studies subsequently documented reduced immune competence in mice, chickens, and cats infected with murine, avian, and feline retroviruses, then still referred to as oncornaviruses (reviewed in [16,17]). In many cases, immunosuppression preceded tumor development, although it was also observed independently of tumor formation.

For instance, feline leukemia virus (FeLV) induces leukemia in only 5–10% of infected cats, yet more than 60% develop opportunistic infections as a result of virus-induced immunosuppression [18]. Indeed, acquired immunosuppression represents the most common and severe outcome of FeLV infection in cats, primarily affecting the cell-mediated arm of the immune system [19]. Koalas have only recently acquired a retrovirus, the Koala retrovirus (KoRV), which is currently in the process of endogenization. Infected individuals often exhibit immunosuppression, making them highly susceptible to opportunistic infections, most notably chlamydiosis, and some develop lymphoma [20].

## 3. Inactivated Retroviruses and Their TM Proteins Are Immunosuppressive In Vitro

### 3.1. Inactivated Retroviruses

When cats were immunized against feline sarcoma virus (FeSV) using either a combined vaccine consisting of killed tumor cells expressing FeSV antigens together with ultraviolet (UV)-inactivated FeLV, or killed tumor cells alone, a higher tumor incidence was observed upon FeSV challenge in the group receiving the combined vaccine. This finding indicated that the antitumor immune response was impaired by the inactivated FeLV [21,22]. Importantly, it demonstrated that active infection is not required for the immunosuppressive effects of retroviruses.

Following these observations, numerous in vitro studies showed that inactivated, non-replicating gammaretroviruses can suppress a range of immune responses, including mitogen-induced proliferation of peripheral blood mononuclear cells (PBMCs) and splenocytes (Table 1). Notably, this immunosuppressive activity appears to be species-independent: murine retroviruses inhibit both mouse splenocytes and human PBMCs [23], and porcine endogenous retroviruses (PERVs) suppress human PBMC responses [24].

### 3.2. Transmembrane Envelope (TM) Proteins

**Figure 1:**
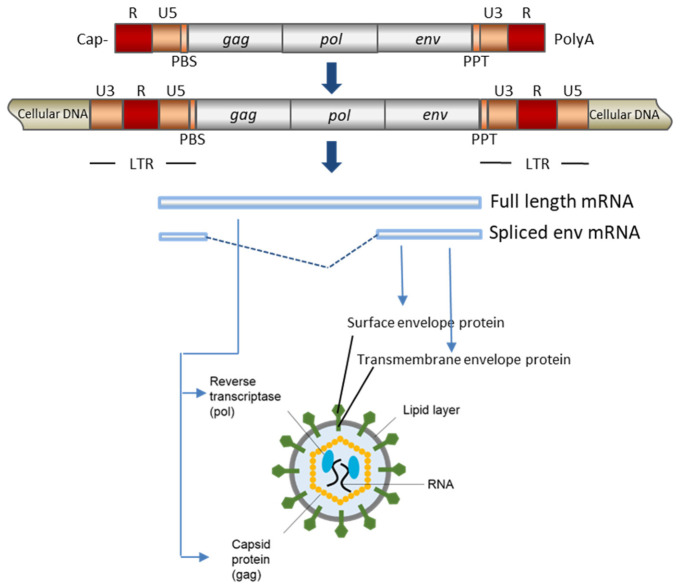
Schematic presentation of the viral genome, the integrated provirus, the transcribed mRNAs, the translated proteins and the virus particle (from top to bottom). The genome consists of 9 Kb of RNA with a 5′ cap and a 3′ polyA tail. The viral RNA is structured as follows: R, a 5′ and 3′ direct repeat; U5 and U3, noncoding unique regions; PBS, a primer binding site; open reading frames for group-specific antigen (*gag*), the capsid proteins, polymerase (*pol*), and envelope (*env*) genes; and PPT, a polypurine tract. The integrated DNA provirus harbors two long terminal repeats (LTRs). From the provirus a full-length mRNA encoding the reverse transcriptase with integrase and protease as well as the capsid proteins and a spliced mRNA encoding the surface (SU) and TM envelope proteins are transcribed. The proteins were incorporated into the virus particle, and the envelope proteins in the lipid membrane. Two copies of genomic RNA, each associated with capsid proteins, are packaged in the virion.

The observation that active infection is not required for retrovirus-induced immunosuppression suggested that a viral protein mediates this effect. To identify the responsible factor, the structural proteins of FeLV were fractionated and tested for their ability to inhibit mitogen-induced proliferation of feline PBMCs. Among these, only the TM protein p15E exhibited immunosuppressive activity, whereas the core protein p27 and other viral proteins were inactive [44]. Retroviral TM proteins are encoded by the env gene and translated from a spliced mRNA. Together with the surface envelope (SU) protein, they form the envelope glycoprotein complexes that appear as protruding spikes on the viral surface (Figure 1). These structures play a critical role in receptor binding and viral entry into host cells.

TM envelope proteins from various retroviruses have been shown to inhibit the function of multiple immune cell types, as well as to modulate cytokine secretion and gene expression in PBMCs [45,46] (Table 2). In addition, the FeLV TM envelope protein p15E suppresses concanavalin A (Con A)-induced receptor capping [47]. Notably, reduced receptor mobility has also been observed in lymphocytes derived from leukemic cats [48], further supporting a role for TM envelope proteins in altering immune cell membrane dynamics.

The TM protein p15E of FeLV inhibited not only the immunoreactivity of feline lymphocytes, but also human, canine, and bovine lymphocytes [59], the TM envelope protein of human endogenous retrovirus-K (HERV-K) inhibited human and murine PBMCs [39].

The TM proteins modulated cytokine expression with marked increase in IL-10 and IL-6 release. Furthermore, a cytokine array analyzing the effect of the TM protein of HERV-K produced in yeast on the expression of 62 cytokines detected an overexpression of the following cytokines: IL-6, IL-8, IL-10, MCP-1, RANTES, MIP-1α, MIP-1β, urokinase-type plasminogen activator receptor (uPAR), soluble tumor necrosis factor receptor type II (sTNFRII), and GCSF [39]. uPAR is a cell-surface receptor on monocytes and macrophages, and its ligand, the serine-protease uPA, acts proteolytically on the cell membrane, degrading surrounding extracellular material (ECM). In addition, uPAR bind with high affinity vitronectin, formyl-peptide receptors, and integrins to activate signaling pathways [60]. sTNFRII is a soluble variant of the extracellular domain of the TNF receptor II, which is derived from the membrane-bound form by the proteolytic activity of a metalloproteinase. sTNFRII is still able to bind to TNF and acts as natural inhibitor of TNF by sequestering soluble TNF and preventing it from binding to its receptor [61].

The HERV-K TM protein was shown to upregulate more than 300 genes and downregulate more than 300 genes. Matrix metalloproteinase-1 (MMP-1), IL-6, IL-1α, and CXCL13 were the genes with the highest upregulation, while selenoprotein P plasma 1 (SEPP1), ficolin 1 (FCN1), dehydrogenase/reductase SDR family member 9 (DHRS9), and FCN2 were those with the highest downregulation [39]. The most elevated expressed gene is MMP-1, a zinc-dependent protease capable of degrading all kinds of extracellular matrix proteins, e.g., in the cleavage of cell surface receptors. The second is IL-6, which correlates with the cytokine studies. The third is IL-1α, which activates lymphocyte proliferation, binding to the interleukin-1 receptor; it activates TNF-α [62]. CXCL13 is selectively chemotactic for B cells; it binds to its receptor, the G-protein coupled receptor (GPCR) CXCR5, and builds a signaling network that regulates not only normal organisms but also the development of many diseases. SEPP1 and FCN1 and 2 are proteins involved in innate immunity. DHRS9 expression distinguishes human regulatory macrophages (Mregs) from a panel of other macrophage subsets [63]. Human Mreg has emerged as a promising cell type used as a cell-based immunosuppressive therapy in solid organ transplant recipients. Pig Mregs were generated from pig CD14^+^ monocytes using macrophage colony-stimulating factor (M-CSF) and IFN-γ, as they secrete IL-10 and TGF-β, suppress T-cell proliferation, and induce FOXP3^+^ regulatory T cells; it has been proposed to use them to protect xenotransplants [64].

Trimeric recombinant ectodomain of HIV-1 gp41 containing the isu domain was produced in human 293 cells under endotoxin-free conditions [57]. Based on antibody-binding studies, the recombinant protein formed six-helix bundles and significantly induced IL-10 production in human PBMCs. Cytokine profiling performed with this trimeric gp41 preparation and compared in parallel with LPS stimulation revealed that both gp41 and LPS induced a range of cytokines and inflammatory mediators. These included MCP-1 (CCL2), CXCL5, IL-8, GRO-α (CXCL1), MIP-1α/MIP-1β, MCP-3 (CCL7), MMP-9, IL-6, and RANTES (CCL5). However, several factors were induced exclusively by gp41, including serpin E1 (plasminogen activator inhibitor-1/nexin), interleukin-1 receptor antagonist (IL-1Ra), uPAR (CD87), platelet-derived growth factor (PDGF-AA), thrombospondin-1 (THBS-1), myeloperoxidase (MPO), and osteopontin (OPN) [57]. These findings demonstrate that HIV-1 gp41 and LPS share overlapping effects on cytokine modulation but also trigger distinct immunological responses. In contrast, LPS, but not gp41, induced upregulation of chitinase-3-like protein 1 (CGP-39) and MIP-3α (CCL20), as well as downregulation of soluble CD14, a component of the LPS receptor complex [57]. Overall, these observations highlight that although gp41 and LPS activate partially overlapping inflammatory pathways, their effects on cytokine regulation are distinct.

## 4. The Immunosuppressive (isu) Domain

**Figure 2:**
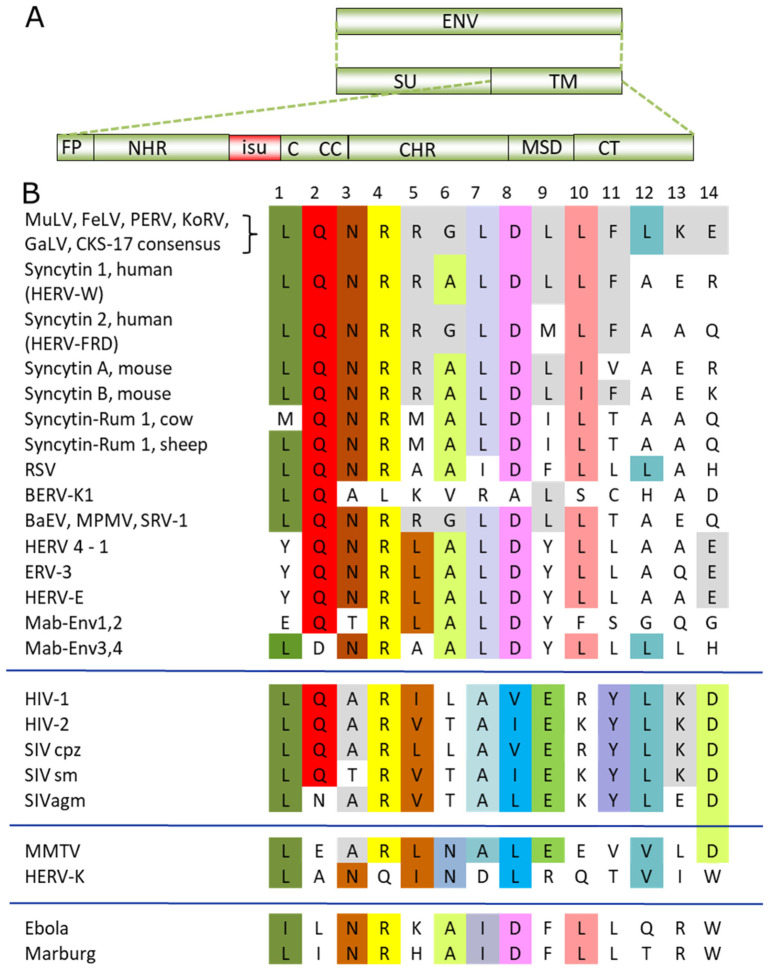
Schematic presentation of retroviral envelope proteins and comparison of the sequences of the immunosuppressive domains of different exogenous and endogenous retroviruses. (**A**), the SU and TM envelope protein were produced from a precursor protein (ENV), the main domains of the TM protein are the fusion peptide (FP), N-terminal helical heptad-repeat region (NHR), the immunosuppressive domain (isu), the cysteine loop (C-CC/C-C), the C-terminal heptad-repeat helix (CHR), the membrane spanning domain (MSD) and the cytoplasmic tail (CT). (**B**), a sequence alignment of the following retroviral sequences was performed: MuLV, murine leukemia virus; FeLV, feline leukemia virus; PERV, porcine endogenous retrovirus; KoRV, koala retrovirus; GaLV, gibbon ape leukemia virus; CKS-17 consensus sequence of the gammaretroviruses; HERV-W, HERV-FRD, human endogenous retrovirus W, FRD; RSV, Rous sarcoma virus, BERV-K1, bovine endogenous retrovirus K1; BaEV, baboon endogenous retrovirus; MPMV, Mason Pfizer monkey virus; SRV-1, simian retrovirus 1; HERV4-1, human endogenous retrovirus 4-1; ERV-3, endogenous retrovirus 3; HERV-H, human endogenous retrovirus-H; Mab-Env1-4, syncytins of Mabuya lizards; HIV-1, -2, human immunodeficiency virus -1, -2; SIV cpz, simian immunodeficiency virus chimpanzee; SIV sm, SIV sooty mangabey; SIVagm, SIV African green monkeys; MMTV, mouse mammary tumor virus; HERV-K, human endogenous retrovirus-K; Ebola virus; and Marburg virus. Identical amino acids and conservative exchanges (L = V = I) in all virus groups or in a single group are stained. The colors highlight identical amino acids at a given position, illustrating the degree of conservation.

The isu domain was first identified using peptides derived from highly conserved regions of the TM envelope protein of gammaretroviruses, including the human T-cell leukemia/lymphoma viruses (HTLVs). One of these peptides, a 17-amino-acid sequence termed CKS-17, was shown to inhibit the proliferation of an interleukin-2-dependent murine cytotoxic T-cell line, as well as the alloantigen-stimulated proliferation of both murine and human lymphocytes [65]. In contrast, four additional peptides representing other regions of the viral protein exhibited no inhibitory activity. The domain corresponding to CKS-17 is 100% conserved among several related gammaretroviruses, including MuLV, FeLV, PERV, KoRV, and gibbon ape leukemia virus (GaLV) (Figure 2).

The isu domain follows the N-terminal helical heptad-repeat region NHR), but it is generally located within the intervening linker/Cys-Cys loop region rather than forming a separate long α-helix. It precedes the C-terminal heptad-repeat helix (CHR), which together with the N-terminal helix forms the characteristic six-helix bundle [66]. This sequence is highly conserved across retroviruses (Figure 2). Notably, this motif has been identified in human syncytin-1 and syncytin-2 (see Section 11), which originated approximately 25 and 40 million years ago, respectively, as well as in HEMO, an envelope (Env) protein encoded by a human endogenous retrovirus dating back around 100 million years [67,68,69]. These findings highlight the ancient evolutionary origin of this motif. A similar sequence has also been described in the retroviral *env*-derived percomORF locus of ray-finned fish, estimated to be approximately 110 million years old [70]. Functional studies using replication-competent MuLV have shown that mutations introduced into the isu domain abolish infectivity, indicating that such mutants are not suitable for in vivo investigation [71,72].

Similar sequences were also found in the envelope protein of filoviruses such as the Ebola virus [73] and the Marburg virus [74] (Figure 2). Ebolavirus causes pronounced immune dysregulation in vivo, combining functional immunosuppression of the adaptive immune response (e.g., lymphocyte loss and impaired antibody production) with simultaneous hyperinflammatory activation [75]. A similar sequence was also found in Nef of HIV-1 [76]. Nef contributes to AIDS pathogenesis by impairing both the humoral and cellular (innate and adaptive) immune responses; it downregulates CD4 and MHC-I.

In numerous experiments, the inhibitory effect of the isu peptide of gammaretroviruses (Table 3) and HTLV, HIV, and the Visna virus (Table 4) on different in vitro immune reactions and on the function of immune cells was demonstrated.

It is important to underline that the isu peptides were not immunosuppressive as monomers; they are only immunosuppressive when conjugated to bovine albumin (BSA) using 1-ethyl-3-(3-dimethylamonopropyl) carbodiimide hydrochloride (EDC) [50,65,77] or in the form of homopolymers cross-linked using EDC or Sulfo-NHS [77]. This indicates that a certain conformation or multiplicity is required in order to interact with the receptor or receptor subunits. This also explains why in situations where monomers or extended monomers were analyzed, no immunosuppressive activities were observed [78].

The importance of molecular conformation is highlighted by the observation that a p15E synthetic peptide conjugated to BSA exhibits immunosuppressive activity that is 10-fold lower than that of the recombinant monomeric form of p15E, and 1000-fold lower than that of the native multimeric p15E complex [50]. Furthermore, the dose of the HIV-1 isu peptide required to induce immunosuppression in vivo is considerably lower than the dose needed to achieve inhibitory effects in vitro [79]. Specifically, systemic administration of as little as 4 pg significantly reduced PMA-induced macrophage recruitment into the peritoneal cavity, an assay that models delayed-type hypersensitivity. This observation is consistent with previous findings showing that as little as 0.2 ng of native p15E is sufficient to exert inhibitory effects in an in vivo assay system [80].

**Table 3:** Influence of the gammaretroviral isu peptides on immune reactions and immune cells in vitro.

Inhibited/Activated Reaction	References
Inhibition of natural killer cells	Harris et al., 1987 [81]
Inhibition of cytotoxic T lymphocytes	Ogasawara et al. 1990 [82]
Inhibition of macrophage-mediated tumor lysis	Kleinermann et al., 1987 [83]
Inhibition of the respiratory burst of monocytes	Harrell et al., 1986 [84]
Inhibition of IgG production	Mitani et al., 1986 [85]
Inhibition of proliferation of interleukin (IL) 2 dependent T cells, inhibition of alloantigen-stimulated proliferation of lymphocytes	Cianciolo et al., 1985 [65]
Inhibition of IL-2-dependent CTLL-2 cells	Ruegg et al., 1989 [50]
Inhibition of delayed-type hypersensitivity in vivo	Nelson et al., 1989 [86]
Inhibition of IL-2 release by mitogen-stimulated EL4 cells	Nelson et al., 1990 [87]
Inhibition of IFN-γ	Ogasawara et al., 1988 [88], Haraguchi et al., 1992 [89]
Modulation of Th1- and Th2-related cytokine mRNA expression: downregulation of IL-12, IFNγ, IL-2, TNF-α, up-regulation of IL-4, IL-5, IL-6 and IL-10 *	Haraguchi et al., 1992 [90], Haraguchi et al., 1993 [91], Haraguchi et al., 1995 [92]
Activation of intracellular cyclic adenosine monophosphate (cAMP)	Haraguchi et al., 1995 [93]
Activation of extracellular signal-regulated kinase 1 and 2 (ERK1/2)	Takahashi et al., 2001 [94]
Activation of mitogen-activated protein kinase/ERK kinase (MEK)	Takahashi et al., 2001 [94]
Activation of protein kinase D (PKD)	Luangwedchakarn et al., 2003 [95]
Activation of Raf1	Fan et al., 2005 [96]
Activation of phospholipase C (PLC) c1	Fan et al., 2005 [96]
Inhibition of protein kinase C	Gottlieb et al., 1990 [97]
Inhibition of LPS/PHA-stimulated cytokine responses, inhibition of TNF-α, CXCL10 and IFN-γ	Tolosa et al., 2012 [58]
Phosphorylation of ERK1 and ERK2, inhibition of TNF-α at 24 h	Lokossou et al., 2020 [98]

* Human PBMCs had been stimulated with lipopolysaccharide (LPS), or Staphylococcus enterotoxin A (SEA).

Similarly to the TM envelope proteins, isu peptides inhibit numerous in vitro immune reactions, including mitogen-induced proliferation of PBMCs and suppression of IgG production. Activation of IL-10 and IL-6 expression as well as reduction of IL-2 secretion are the main effects both of CKS-17 [92] and HIV-1 isu [77]. Particularly noteworthy are findings regarding their effects on signal transduction pathways. The isu peptide CKS-17 of gammaretroviruses has been shown to activate cAMP [93], ERK1/2, and MEK [96], and inhibit protein kinase C [95,96,97] (Table 3). CKS-17 is capable of inducing phosphorylation of PLCγ, MEK, ERK1/2, and protein kinases C and D [95,96,98] which are involved in signal transduction via the Ras-Raf-MEK-MAPK and PI3K-AKT-mTOR pathways [99].

**Table 4:** Immunosuppressive properties of synthetic isu peptides derived from HTLV, HIV, and Visna virus in vitro (see Table 3 for isu peptides of gammaretroviruses).

Virus	Inhibited Reaction	Reference
HTLV, HIV	Inhibition of stimulation by anti-CD3 antibody and inhibition of MLR	Cianciolo et al., 1988 [100]
HIV-1	Inhibition of mitogen stimulated proliferation of human PBMCs	Ruegg et al., 1989 [101]
HIV-1	Inhibition of lymphocyte activation, protein kinase C and intracellular calcium influx	Ruegg et al., 1990 [102], Ruegg et al., 1991 [79]
Visna virus	Inhibition of lymphoproliferation and protein kinase C	Ruegg et al., 1990 [103]
HIV-1	Inhibition of lymphoproliferation	Denner et al., 1994 [104]
HIV-1	Inhibition of T and B cell proliferation	Denner et al., 1996 [105]
HIV-1	Single mutations abrogate immunosuppressive properties	Morozov et al., 2012 [106]
HIV-1	Modulation of cytokine and gene expression	Denner et al., 2013 [77]
HIV-1	Binding to monocytes and T cells	Mühle et al., 2016 [107]

Similar to the isu peptide of p15E [97], the isu peptides derived from HIV, HTLV, and Visna virus have been shown to inhibit protein kinase C (PKC) activity [102,103]. PKC regulates the function of numerous proteins through phosphorylation and plays a central role in multiple signal transduction pathways. Notably, the HIV-1 isu peptide suppresses both PKC-dependent IL-2 production and the Ca^2+^ influx-dependent, yet PKC-independent, induction of IL-2 receptor expression [79].

Mutations within the isu domain of HIV-1 have been shown to abolish its immunosuppressive activity [106]. As illustrated in Figure 2, the first four amino acids are the most highly conserved within the sequence. Substitutions at these positions, as well as at residues 9 and 14, completely eliminated the observed effect.

Using a cytokine array, 16 cytokines were identified as upregulated following treatment of human PBMCs with homopolymers of the HIV-1 isu peptide: IL-1β, IL-6, IL-8, IL-10, IL-13, GM-CSF, MCP-1, MCP-2, MDC, MIP-1α, MIP-1β, MIP-3α, RANTES (CCL5), TNF-α, IFN-γ, and GRO. In contrast, IL-2 and CXCL9 were downregulated [77]. These results are in agreement with the results obtained with the TM protein of HERV-K [39] (see above). In HIV-1 infected patients, elevated IL-6 levels [108,109] and an elevation of IL-10, TNF-α, and IFN-γ [110] was reported.

Cytokine ELISA analyses showed that IL-10 release into the supernatant of human PBMCs treated with the polymeric isu peptide peaked at 15 h, whereas IL-10 mRNA expression reached its maximum at 10 h. Notably, the magnitude of IL-10 production varied among different blood donors [77].

Comparative RNA sequencing of PBMCs treated with the HIV-1 isu peptide versus untreated controls revealed that IL-6, MMP-1, IL-1α, and CXCL5 were among the most 300 upregulated genes, while FCN1, DHRS9, SEPP1, and TREM2 were among the most 300 downregulated [77]. These findings are consistent with results obtained using the recombinant HERV-K protein [39] (see above). For IL-1α and TNF-α, variable alterations in serum levels in HIV-1-infected patients were found: there was a positive correlation between serum levels of TNF-α and IL-1α in nearly all patients. Detectable levels of TNF-α and IL-1α were found in 34% of asymptomatic patients and only rarely detectable in AIDS patients. There was a positive correlation between serum levels of both cytokines [111]. When monocyte-derived macrophages (MDMs) from normal donors were infected with HIV-1, CXCL5 was upregulated significantly both at the mRNA and protein level [112].

## 5. Quantitative Analysis of the Immunosuppressive Effects

A direct comparison of the immunosuppressive potency of different retroviruses is challenging because the available studies used heterogeneous experimental systems, including different assays, preparations (intact virus versus recombinant proteins versus conjugated peptides), and readouts. Nevertheless, the reported effects appear to be within a comparable range, with proteins generally showing higher activity than corresponding isu peptides due to conformational requirements.

For example, 50 µg/mL of an HIV-1 isu peptide homopolymer induced an IL-10 release of 750 pg/mL in 3 × 10^5^ human donor PBMCs [77]. Similarly, 12.5 µg/mL of recombinant HERV-K protein induced an IL-10 release of 850 pg/mL [39], while co-incubation of 3 × 10^5^ human donor PBMCs with 3 × 10^5^ cells expressing PERV p15E resulted in an IL-10 release of 500 pg/mL [113]. Consistent with the importance of conformation, recombinant HIV-1 gp41 was reported to be approximately 700-fold more potent than homopolymers of the corresponding isu peptide in inducing IL-10 release [106]. In addition, high-molecular-weight MuLV p15E complexes inhibited IL-2-driven proliferation of a murine T-cell line with an ID_50_ of ~10 µg/mL, whereas recombinant p15E showed activity at an ID_50_ of ~30 µg/mL [50].

In vivo studies in mice demonstrated that retroviral immunosuppressive effects can occur at very low doses. For instance, 4 µg of the HIV-1 isu peptide significantly reduced PMA-induced macrophage recruitment into the murine peritoneal cavity [79], while as little as 0.2 ng of native p15E inhibited macrophage accumulation during delayed inflammatory responses [80].

## 6. Sequence Homology with Interferons

Sequence homology has been identified between the isu domains of retroviruses and interferon sequences [114] (Table 5). Consistent with this observation, a synthetic peptide corresponding to a 10-amino-acid region (residues 9–18) of interferon-α (IFN-α) was shown to inhibit both the proliferation of the Daudi lymphoblastoid cell line and antigen receptor-stimulated proliferation of primary human T lymphocytes [114]. The same inhibitory effect was observed with the gammaretroviral isu peptide CKS-17 (Table 3). Interestingly, two regions within IFN-α display similarity to retroviral isu domain sequences: one located in the N-terminal region (residues 9–24 in the case of α-1 IFN), resembling isu sequences found in gammaretroviruses, and a second in the C-terminal region (residues 113–124 in the case of α-1 IFN), which is more closely related to isu sequences from immunodeficiency viruses such as HIV-1.

Type I interferons signal through two receptors, IFNAR1 and IFNAR2, and the isu-related regions overlap only partially with the known receptor-binding sites. Although it has been demonstrated that type I interferons can exert immunosuppressive effects, including the induction of IL-10 release (reviewed in [115]), it remains unclear whether these effects involve interactions with additional, as yet unidentified, receptors.

Antibodies against human IFN-α and -β recognized gp41 and inhibited gp41-binding to a potential cellular receptor protein p45 [116,117]. In this context, it may be of interest that IFN-α-vaccine-treated HIV-infected patients showed a significant reduction of disease progression, which was associated with an anti-IFN-α antibody response and increase of its IFN-α-neutralizing capacity, and that these anti-IFN-α antibodies cross-reacted with the isu domain [118]. Human IFN-β, after preincubating with cells, could incompletely inhibit the binding of gp41 to human B cells and monocytic cells, suggesting that IFN-β and gp41 of HIV-1 may use the same receptor [116].

**Table 5:** Sequence homology: Immunosuppressive domains of retroviruses and different IFNs. Amino acids identical to the isu sequence of gammaretroviruses are in red, amino acids identical to the isu sequence of HIV-1 are in light blue. HIV-1, human immunodeficiency virus -1; FeLV, feline leukemia virus; MuLV, murine leukemia virus; PERV, porcine endogenous retrovirus; TP, trophoblast protein; ref., references.

Protein/Virus	Sequence (Amino Acids)																	Ref.
FeLV, MuLV, PERV	** L **	** Q **	**N**	** R **	** R **	**G**	** L **	**D**	** L **	** L **	**F**	** L **	**K**	**E**	** G **	** G **	** L **	[65]
HIV-1	** L **	** Q **	**A**	** R **	**I**	**L**	**A**	**V**	**E**	**R**	**Y**	** L **	**K**	**D**	**Q**	**Q**	** L **	[104]
	N-terminal IFN sequence																	
human α-7 IFN	** L **	**R**	** N **	** R **	** R **	**A**	** L **	**I**	** L **	** L **		**A**	**Q**	**M**	** G **	**R**	**I**	[119]
murine α-1 IFN	** L **	**R**	** N **	**K**	** R **	**A**	** L **	**T**	** L **	** L **		**V**	**Q**	**M**	**R**	**R**	** L **	[120]
human α-1 IFN	** L **	**D**	** N **	** R **	** R **	**T**	** L **	**M**	** L **	** L **		**A**	**Q**	**M**	**S**	**R**	**I**	[121]
human α-C IFN	** L **	**G**	** N **	** R **	** R **	**A**	** L **	**I**	** L **	** L **		**G**	**Q**	**M**	** G **	**R**	**I**	[122]
human α-2 IFN	** L **	**G**	**S**	** R **	** R **	**T**	** L **	**M**	** L **	** L **		**V**	**Q**	**M**	**R**	**K**	**I**	[123]
bovine α-2 IFN	**L**	**V**	**G**	**R**	**Q**	**N**	**L**	**R**	**L**	**L**		**G**	**Q**	**M**	**R**	**R**	**L**	[124]
porcine α -1 IFN	**L**	**A**	**H**	**T**	**R**	**A**	**L**	**R**	**L**	**L**		**A**	**Q**	**M**	**R**	**R**	**I**	[125]
human ω-1 IFN	**L**	**L**	**S**	**R**	**Q**	**T**	**L**	**V**	**L**	**L**		**H**	**Q**	**M**	**R**	**R**	**I**	[126]
ovine TP-1	**L**	**D**	**A**	**R**	**E**	**N**	**L**	**K**	**L**	**L**	**D**	**R**	**M**	**N**	**R**	**L**	**X**	[123]
ovine/bovine TP-1	**L**	**Q**	**D**	**R**	**K**	**D**	**F**	**G**	**L**	**P**	**Q**	**E**	**M**	**V**	**E**	**G**	**D**	[127]
ovine TP-1	**L**	**K**	**D**	**R**	**R**	**D**	**F**	**R**	**F**	**P**	**Q**	**E**	**M**	**V**	**K**	**G**	**S**	[128]
human β-1 IFN	**L**	**N**	**G**	**R**	**L**	**E**	**Y**	**C**	**L**	**K**	**D**	**R**	**M**	**N**	**F**	**D**	**I**	[122]
HIV-1	**L**	**Q**	**A**	**R**	**I**	**L**	**A**	**V**	**E**	**R**	**Y**	**L**	**K**	**D**	**Q**	**Q**	**L**	[104]
	C-terminal IFN sequence																	
human α-1 IFN	**N**	**A**	**D**	**S**	**I**	**L**	**A**	**V**	**K**	**K**	**Y**	**F**	**R**	**R**	**I**	**T**	**L**	[121]
human IFN-α 4, 7, 10, 14, 16, 17	**N**	**E**	**D**	**S**	**I**	**L**	**A**	**V**	**R**	**K**	**Y**	**F**	**Q**	**R**	**I**	**T**	**L**	[129]

## 7. Transmembrane (TM) Envelope Proteins Are Immunosuppressive In Vivo

Following these in vitro findings demonstrating the immunosuppressive properties of retroviral TM envelope proteins, in vivo studies were required to confirm their physiological relevance (Table 6). First evidence for their in vivo activity was obtained when cats were immunized against the feline sarcoma virus (FeSV) using either a combined vaccine consistent of ultraviolet (UV)-inactivated FeLV preparations and killed tumor cells expressing FeSV antigens or killed tumor cells alone. An increased tumor incidence was observed upon challenge with FeSV in the first group, indicating that the antitumor immunity was inhibited by the inactivated FeLV [21,22].

Similar observations were made with purified p15E from feline leukemia virus (FeLV) [44]. Groups of cats were immunized with either killed tumor cells expressing the feline oncornavirus-associated cell membrane antigen (FOCMA) or with these tumor cells in combination with FeLV p15E. Following challenge with feline sarcoma virus (FeSV), three of four cats receiving p15E developed progressive, fatal fibrosarcomas compared to one of five not treated with p15E. In addition, the p15E-treated cats exhibited lower antibody titers against FOCMA, indicating that p15E suppressed the antibody response to this tumor-associated antigen and increased tumor susceptibility after FeSV challenge [44].

Consistent with these findings, low-molecular-weight proteins, most likely p15E, from various murine retroviruses inhibited the accumulation of macrophages at sites of delayed-type inflammatory reactions when administered to mice [80].

**Figure 3:**
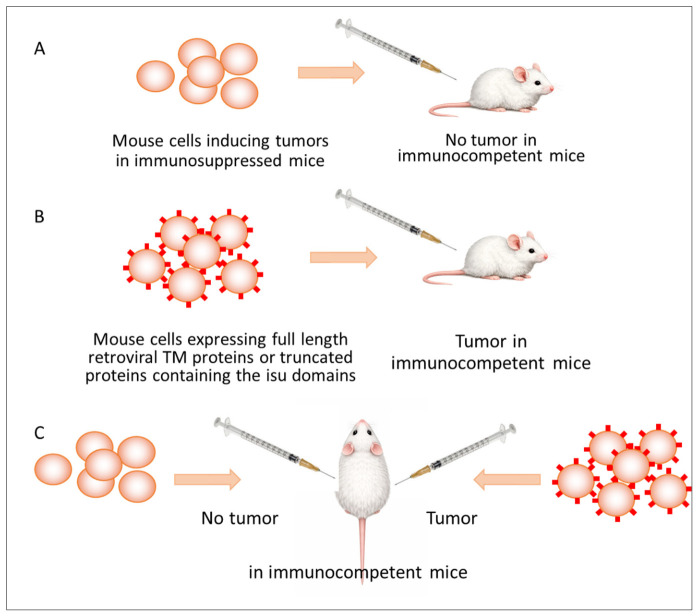
Effect of the expression of retroviral TM envelope proteins on mouse tumor cells, which produce tumors in X-irradiated or SCID mice, but not in immunocompetent mice on tumor formation in immunocompetent mice (**A**). TM proteins or truncated TM proteins containing only the isu domain from several retroviruses, including MuLV, HERV-H, MPMV, human syncytin-2, and mouse syncytin-B, were found to exhibit immunosuppressive activity in this assay [130,131,132,133,134,135] (**B**). Notably, when untreated tumor cells were injected into one flank of an immunocompetent animal, no tumors developed; however, injection of tumor cells expressing a retroviral TM protein into the opposite flank led to tumor formation [130] (**C**).

The laboratory of Thierry Heidmann developed a tumor rejection assay that was used to demonstrate the immunosuppressive properties of the TM envelope proteins of numerous retroviruses [130,131,132,133,134,135] (Figure 3). This assay is based on mouse tumor cells that fail to form tumors in immunocompetent mice but readily do so in X-irradiated or SCID mice. It was initially applied to study the p15E protein of murine leukemia virus [130]. Subsequently, TM proteins from HERV-H [132] and MPMV [131], as well as human syncytin-2 and murine syncytin-B [133] (see Section 11), were shown to exhibit immunosuppressive activity in this system, whereas human syncytin-1 and murine syncytin-A did not. When full-length and truncated/deleted TM envelope proteins of the MoMuLV were expressed on the surface of tumor cells, it was demonstrated that the isu domain alone was sufficient to confer immunosuppressive activity [133]. Moreover, targeted amino acid substitutions enabled the conversion of an immunosuppressive syncytin into a non-immunosuppressive form, and vice versa [133].

Within the framework of these studies, a F-MuLV carrying mutations in the isu domain was generated. This immunosuppression-deficient virus retained in vitro infectivity comparable to that of the wild-type virus. Importantly, this mutant demonstrated that the isu domain suppresses both innate immune responses, including natural killer (NK) cell activity, and adaptive immune responses mediated by CD8^+^ T cells.

## 8. Immunosuppressive Properties of Cell-Associated TM Proteins In Vitro

Since a few previous studies using recombinant gp41 produced in bacteria [53,54,55,58] or conjugated peptides could not rule out endotoxin contamination below the detection limits of the used detection assays, it is essential to ensure rigorously verified endotoxin-free conditions. Endotoxin is a lipopolysaccharide which binds to TLR4 resulting in the release of cytokines, among them IL-10 [136]. To investigate the activity of retroviral TM envelope proteins under endotoxin-free conditions, recombinant proteins were expressed either in yeast systems [39] or in human cells cultured under endotoxin-free conditions [57]. In these studies, activation mediated by LPS binding to the TLR4/CD14/MD-2 receptor complex could be excluded, as the protein preparations were demonstrated to be free of LPS. Differences in the cytokine modulation between HIV-1 gp41 and LPS are described in Section 3.

An alternative approach to studying retroviral TM envelope protein activity under endotoxin-free conditions was the expression of these proteins on the surface of cells maintained in endotoxin-free culture systems [57,113]. For example, when murine cTRAMP prostate cancer cells were transfected with a vector expressing the ectodomain of HIV-1 gp41 without the fusion peptide, surface expression of gp41 was confirmed by flow cytometry (FACS), and the protein was also detected in the culture supernatant [57] (Table 7). Upon pulsing these cells with the ovalbumin-derived MHC class I peptide SIINFEKL and co-culturing them with naïve CD8^+^ T cells from OT-1 mice, which express a SIINFEKL-specific T cell receptor, a marked inhibitory effect was observed. Specifically, gp41-expressing cells—but not vector control cells—strongly suppressed IFN-γ production and reduced CD25 (IL-2 receptor) expression. These results demonstrate that ectodomain of gp41 containing the isu domain impairs antigen-specific responses of murine CD8^+^ T cells, primarily through potent suppression of IFN-γ production [57].

Similarly, human 293T cells were transfected with vectors expressing the TM envelope protein p15E of porcine endogenous retrovirus (PERV). When these cells were co-incubated with PBMCs from healthy donors, cytokine analysis revealed increased production of IL-10 and IL-6 compared to PBMCs co-incubated with control 293T cells lacking p15E expression [113] (Table 7). A comparable increase in IL-10 and IL-6 was also observed when PBMCs were co-incubated with 293T cells producing PERV that naturally expressed p15E. In addition, elevated levels of MMP1 were detected.

Importantly, p15E expression conferred protection against cellular cytotoxicity. Moreover, p15E-expressing 293T cells exhibited reduced surface expression of MHC class I molecules [113]. This finding is consistent with the well-established strategy of retroviruses to evade cytotoxic T lymphocyte-mediated killing through downregulation of MHC expression, as previously demonstrated for HIV-1 [137]. In addition, downregulation of MHC class II molecules has also been reported in infections with murine retroviruses closely related to PERV [138].

Whereas PERV p15E expressed on human 293 cells, HIV-1 and MuLV downregulated the expression of MHC class I molecules [113,137]; other reports showed that recombinant gp41 as well as type I interferons IFN-α and -β could enhance cell surface expression of human MHC I and II molecules [117]. The causes underlying this difference require further investigation.

## 9. Potential Receptor Molecules

Cell surface proteins binding to the isu peptide of HIV-1 and to p15E of gammaretroviruses have been described that may represent receptors [107,139,140,141,142,143,144,145,146,147]; however, their identity is still unknown. The isu peptide of HIV-1 coupled to human serum albumin was shown to bind to CD4^+^ cells and a 45 kDa binding protein was isolated [139]. Later, in addition to the 45 kDa protein, an 80 kDa (p80) protein was detected on human CD4^+^, whereas on murine P815 cells, only the 80 kDa protein was detected [145]. A segment of eight amino acids within the isu peptide contained the minimum sequence able to bind. Both proteins were purified by affinity chromatography and the peptide as well as antibodies against p80 inhibited virus infection, suggesting that this region binds to a receptor involved in infection [145].

Using affinity chromatography with immobilized recombinant gp41 (amino acids 539–684), five gp41-binding proteins of 37, 45, 50, 62, and 100 kDa were isolated from lysates of Raji B-cells. Antisera against the proteins p45 and p62 showed that all proteins were not related [143]. These proteins were also found on human CD4^+^ cells [141,143]. However, recombinant HIV-1 gp41 bound to blood B lymphocytes and monocytes more strongly than to T lymphocytes [146].

Using fluorescence-activated cell sorting and competition experiments, it was shown that homopolymers of the isu peptide of HIV-1 bind specifically to human PBMCs, mainly to monocytes and B cells [107]. When a biotinylated trimeric ectodomain of HIV-1 gp41 without the fusion peptide produced in human 293 cells was co-incubated with freshly isolated PBMCs at low temperatures to prevent nonspecific uptake, detection using fluorescent streptavidin revealed binding to monocytes and, to a lesser extent, to lymphocytes [57].

Previously, coupling of ^125^I-labelled isu peptides with the cross-linker disuccinimidyl suberate (DSS) to the surface of human PBMCs allowed detection of binding proteins with a molecular weight of 40, 60, and 100 kDa [140,147]. Similar results were obtained when affinity chromatography and subsequent polyacrylamide gel electrophoresis (PAGE) were performed with the isu peptide of HIV-1 or the purified recombinant p15E of PERV; in both cases, proteins with a molecular weight of 37, 60, and 100 kDa were isolated [140,147].

^125^I-labeled p15E isolated from FeLV was shown to bind to surface proteins on the surfaces of human PBMCs and Raji, MOLT-4, and U-937 cells [144].

There is also evidence that a subunit of the isu domain of gammaretroviruses, the hexapeptide LDLLFL, may bind to the receptor of N-formyl-methionyl-leucyl-phenylalanine (FMLP) [148]. FMLP is a potent chemoattractant used to stimulate macrophages and neutrophils, and the p15E-derived LDLLFL is a potent inhibitor of the FMLP-induced polarization response that is an early event in chemotaxis of monocytes and granulocytes. LDLLFL inhibited the changes in [Ca^2+^]i in response to FMLP; in the presence of LDLLFL, the FMLP dose-response curve shifted to higher concentrations, indicating that LDLLFL interfered with binding of FMLP to its receptor. Indeed, the binding of [^3^H]FMLP to neutrophilic granulocytes was inhibited in the presence of LDLLFL.

The detection of putative receptors on T cells [107,141,142,144,146], B cells [107,143], monocytes [107], and myeloid cells [144], combined with the inhibitory effects of retroviral TM proteins on T cells [50], B cells [85], natural killer cells [81], neutrophils [33], erythroid colony-forming cells [30], and monocyte cytokine release [53,54,56], suggests that the corresponding receptor is widely expressed among hematopoietic cell populations. Moreover, the broad interspecies activity of retroviral TM proteins and their immunosuppressive (isu) peptides [23,24] supports the notion that these receptors are evolutionarily conserved.

## 10. Implications for Infections with Immunodeficiency Viruses

Immunodeficiency viruses are complex retroviruses encoding accessory and regulatory proteins that control viral replication and contribute to the immunosuppression in vivo. For example, Nef of HIV-1 downregulates CD4 and major histocompatibility complex class I (MHC-I) alters T cell signaling and activation, and enhances viral replication and infectivity [149]. Furthermore, it is well known that the surface envelope protein gp120 of HIV-1 contributes to the AIDS-associated immunosuppression since it binds to CD4^+^ cells. Multiple in vitro studies have demonstrated that HIV-1 gp120 exerts immunosuppressive effects independently of viral infection. Purified gp120 inhibits CD4^+^ T cell proliferation and cytotoxic function, induces immunosuppressive cytokines such as IL-10 in dendritic cells, impairing their maturation and T cell stimulatory capacity, and suppresses B cell proliferation through induction of transforming growth factor-β 1 (TGF-β1) [150,151]. Meanwhile, there is evidence that the surface envelope protein gp70 of F-MuLV also seems to suppress vaccine-induced CD8^+^ T-cell responses mediated by interleukin-10-producing CD4^+^ T cells [152,153]. It will be important to identify the immunosuppressive motifs in the surface envelope proteins.

Nevertheless, the isu domain may also play a crucial role in infections with immunodeficiency viruses. During primary HIV-1 infection, only gp41 and gp120 are available to interact with the immune system of the infected individual, whereas Nef and other accessory proteins are produced only at later stages. During untreated infection, both the viral load and the amount of gp41 continuously increase (see Figure 4 in [45]) and the viral load correlates with disease severity. Since the isu domain is normally shielded by the SU envelope protein in intact virions, the question arises as to how this domain can interact with the immune system and contribute to immunosuppression. The explanation is straightforward: TM envelope proteins with an exposed isu domain can occur in several forms within infected organisms. First, they may be present on the surface of viral particles following shedding of the SU envelope protein. Second, they can be detected on the surface of infected cells during the budding process and after SU shedding. Third, they may occur as components of circulating immune complexes, for example as TM envelope protein–antibody complexes (see Figure 2 in [45]). In the case of HIV-1, viral particles have been shown to contain gp120-depleted gp41 stumps, which are present either as monomeric or trimeric structures [154]. This is certainly also true for gammaretroviruses. As discussed in Section 8, cell-associated retroviral TM envelope proteins exhibit immunosuppressive activity comparable to that of soluble, free-floating proteins.

In the literature, immune cells from HIV-1-infected individuals and SIV-infected monkeys are sometimes described as being “exhausted” [155]. However, the restoration of their functionality following treatment with PD-1-targeting antibodies indicates that these cells are not irreversibly dysfunctional but are instead subjected to active suppression. PD-1 blockade has been shown to reduce excessive immune activation and microbial translocation, which are secondary to HIV-1-induced immunosuppression [156].

It is important to note that even when individuals with HIV-1 receive effective antiretroviral therapy (ART) and achieve suppressed or undetectable plasma viral loads, multiple studies have demonstrated the persistence of immune dysfunction, chronic immune activation, inflammation (including elevated IL-6 levels), incomplete immune reconstitution and CD4^+^ T-cell recovery, as well as ongoing impairment of gut mucosal immunity and microbial translocation [157,158]. Because the amount of free circulating virus is low during effective ART, new rounds of infection are limited. However, persistent HIV-1 infection can be explained by the clonal proliferation of cells that were infected prior to the initiation of antiretroviral therapy [159]. These cells may contain substantial levels of HIV-1 RNA [159,160]. Most importantly, examination of lymph node biopsies from HIV-1-infected individuals after 5–13 months of therapy revealed persistent expression of the envelope glycoproteins gp120 and gp41 within germinal centers, despite suppression of plasma viremia and undetectable levels of HIV mRNA [161]. It is therefore plausible that the immunosuppressive activity of gp41 contributes to the maintenance of immune dysfunction by modulating cytokine release, impairing mucosal immunity, and promoting microbial translocation, which in turn may contribute to chronic immune activation.

## 11. Implications for Reproduction

Retroviral envelope genes have been co-opted, “enslaved” for a role in placentogenesis by numerous lineages of mammals, including eutherians and marsupials, representing a variety of placental structures (for review see [162,163,164]). The presence of the retroviral envelope protein percomORF within the group Percomorpha, unique among spiny-finned fishes in having evolved placentation and live birth, and in viviparous lizards, is especially intriguing [70,165].

The human placenta is a preferential site of HERV expression, as shown by different methods such as RT-PCR, immunocytochemistry, and Western blot analyses. Strongly elevated expression of HERV-W (syncytin-1), HERV-FRD (syncytin-2), and ERV-3 or HERV-R, as well as medium expression of HERV-K and HERV-E in the human placenta, has been reported [166]. Syncytin-1, the envelope protein of HERV-W, and syncytin-2 (HERV-FRD) mediate the fusion of the villous cytotrophoblast in the human placenta to form the multinucleated syncytiotrophoblast.

Despite the similarity in the sequence of the isu domain, syncytin-2 is immunosuppressive, whereas syncytin-1 is not immunosuppressive [133]. Similarly, the murine syncytin-A is not immunosuppressive, whereas murine syncytin-B is immunosuppressive [133]. Using deletion mutants, it was shown that the highly conserved isu domain in the TM protein is responsible for the immunosuppressive effect. Mutations of specific amino acids allowed the switch from an immunosuppressive to a non-immunosuppressive syncytin and vice versa.

Expression of the HERV-K TM protein has also been reported in the human placenta [167], and its immunosuppressive properties have been extensively studied [39] (Table 2).

The presence of placenta cells expressing retroviral envelope proteins surrounded by immune cells deep in the maternal tissue supports a general immunosuppressive function. It is important to emphasize that during evolution, different species utilized, or “enslaved”, different endogenous retroviruses, and that two or more endogenous retroviruses are involved in placentogenesis in some investigated species [164].

In contrast to the findings, where syncytin-1 expressed on the surface of tumor cells was not immunosuppressive in the tumor rejection assay, recombinant syncytin-1 produced in *E. coli* and a dimer of the immunosuppressive peptide of syncytin-1 were found to inhibit LPS/PHA-stimulated cytokine responses and expression of TNF-α, CXCL10, and IFN-γ [58]. The reason for this discrepancy remains unclear. It was shown that syncytin-1 was incorporated into placental exosomes.

Syncytin-2, which was immunosuppressive in the tumor rejection assay, was also shown to be present in the exosomes, and these inhibited the production of Th1 cytokines (TNF-α, IFN-γ, and IL-2) [98]. The placenta, most specifically the syncytiotrophoblast layer, produces an important amount of extracellular vesicles that promote feto-maternal tolerance. By this mechanism, syncytin-2 could modulate the immune response at a distance.

## 12. Implications for Tumor Progression

In the past, p15E-related antigens were described in human malignant and mitogen-stimulated PBMCs [168]. p15E-related proteins were found in numerous spontaneous murine primary tumors, as well as in all murine tumor cell lines tested, suggesting that the expression of such immunosuppressive proteins by transformed cells in vivo could confer a selective advantage for their sustained growth since they would be more likely to escape immune surveillance [169]. p15E-related proteins were described in human colorectal and gastric cancer [170] and human head and neck cancer [171,172], and were also found in the serum of the patients [173]. Open reading frames of HERV-H were shown to contain an isu domain very similar to p15E of murine and feline retroviruses [174] (Table 8). The presence of immunosuppressive proteins cross-reacting with monoclonal antibodies to MuLV p15E in human tumors have suggested a role of HERV Env proteins in immunosuppression in malignant tissue [168,169].

**Figure 4:**
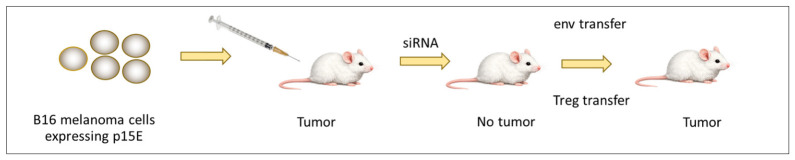
Expression of the TM protein p15E is required for mouse melanoma tumor growth in vivo. Injection of B16 melanoma cells, which express p15E, resulted in tumor development in mice. Knock-down of Env expression by specific siRNA prevented tumor development. Tumor development was restored by transfection of *env* or transfer of Treg cells from tumor bearing mice [175,176].

An endogenous retrovirus specifically produced by a murine melanoma was shown to be required for tumor growth in immunocompetent mice (Figure 4). Knocking down the virus by RNA interference resulted in rejection of the engrafted tumor cells, whereas transfer of env and Treg cells again allowed tumor growth [175,176].

**Figure 5:**
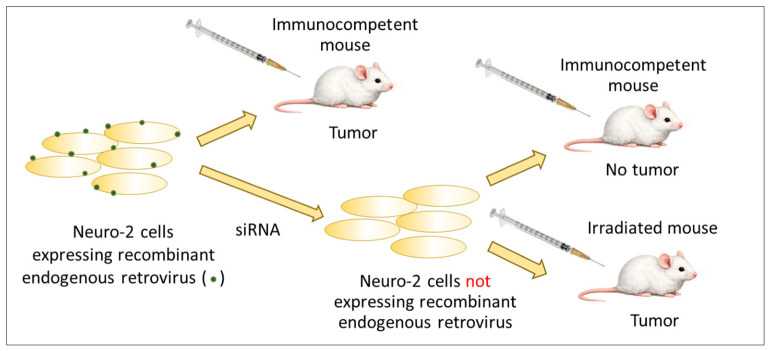
Expression of a recombinant endogenous retrovirus encoding the TM envelope protein p15E is required for tumor development of the Neuro-2 cell line in immunocompetent mice [166,177].

In another tumor model, the Neuro-2 cell line derived from a spontaneous A/J mouse neuroblastoma was shown to express an infectious retrovirus that resulted from a recombination of two endogenous retroviral elements [177] (Figure 5). These tumor cells produced tumors in immunocompetent mice. However, when knocking down the expression of the retrovirus by RNA interference, which did not affect the transformed state of the cell, they were still able to produce tumors in irradiated mice, but not in immunocompetent mice. This indicates that an invading tumor can be the result of the recombination of endogenous retroviruses followed by an amplification of this element and high-level expression of the immunosuppressive TM protein [177].

## 13. Implications for Vaccine Development

Immunization with TM proteins mutated in the isu domain induced higher immune responses compared with immunization with the unmutated, immunosuppressive protein. This was first shown for F-MuLV: UV-inactivated F-MuLV with a mutated isu domain induced much stronger humoral and cellular immune responses compared with their wild-type viruses, and induced a stronger induction of IFN-γ by CD4^+^ and CD8^+^ T cells [135]. Similarly, the mutation of the isu domain in a well-characterized canarypox virus-vectored vaccine against FeLV significantly increased the efficacy, increased the frequency of IFN-γ-producing cells, and reduced the frequency of IL-10-producing cells [134].

Immunization of mice with wild-type syncytin-1, which lacks immunosuppressive activity in the tumor rejection assay, elicited a robust IgG antibody response [133]. In contrast, no antibodies were induced when mice were immunized with a mutant syncytin-1 protein rendered immunosuppressive.

Cynomolgus macaques were vaccinated with a measles virus replicative vector expressing simian/human immunodeficiency virus (SHIV) Gag, Env, and Nef antigens, either with wild-type or mutated isu domains of Nef and Env antigens [178]. Nef has been shown to contain a sequence highly related to the isu domain of gammaretroviruses [76]. The inactivation of the isu domains in Nef and Env led to the induction of significantly enhanced cellular immune responses. Vaccinees were better protected against viremia; some were even completely protected after challenge. Mutation of the isu domains clearly increased vaccine immunogenicity and they should be included in HIV vaccine strategies [178].

These studies provide new promising strategies to identify immunosuppressive domains within viral antigens and knock down this function by mutations minimally altering the structure of the antigen in order to generate more efficient vaccines [134].

## 14. Implications for Xenotransplantation

Based on the findings that several TM proteins and the isu domain expressed on the surface of cells protect tumor cells from immunological rejection [130,131,132,133,134,135], the idea was developed that the expression of the TM protein of PERV on the surface of the xenotransplant may protect the transplant from rejection, or might at least reduce pharmacological immunosuppression [113,179,180].

The TM of PERV is the best candidate, since p15E is recognized as a self-antigen and pigs are tolerant against p15E, not producing antibodies [181].

**Figure 6:**
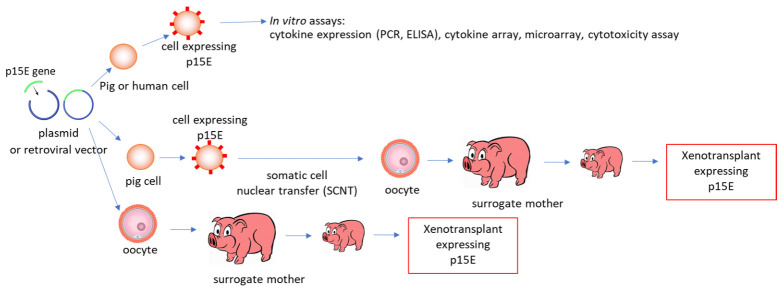
The upper panel illustrates the experimental setup described in [113]. The p15E protein of PERV is expressed on the cell surface, and its effects on cytokine expression and secretion in co-incubated human PBMCs, as well as on cytotoxic cell activity, were analyzed. The lower panel outlines a proposed strategy for generating p15E-expressing xenotransplants in vivo. In one approach, cells expressing p15E are used for somatic cell nuclear transfer (SCNT) to introduce the genetic information into oocytes. Alternatively, the p15E gene may be introduced directly into oocytes. In both approaches, surrogate mothers are required to generate piglets that express p15E systemically across all organs.

As a proof-of-concept experiment, p15E was expressed in 293T cells, and these were incubated with PBMCs from healthy donors. The production of IL-10 and Il-6 increased compared with the PBMCs co-incubated with 293T cells, which did not express p15E [113] (Table 7). Furthermore, increased expression of MMP1 was also observed. Most importantly, it was shown that p15E inhibited cytotoxic cells. An inhibition of CD107a expression in CD45^+^CD56^+^ was observed, which is generally interpreted as an inhibition of NK-cell cytotoxic activity, but also of activated T cells, which are CD56^+^. When the protective effect against cellular cytotoxicity is confirmed, this will be a good indication that p15E can be used to protect the xenotransplant (Figure 6).

The expression of p15E in pigs requires careful evaluation. Ubiquitous expression across all cell types, including immune cells, could potentially impair immune function. One promising strategy to mitigate these risks is the use of organ-specific promoters, which would restrict p15E expression to defined organs, thereby preserving systemic immune competence. Organ-specific promoters are already well established in gene therapy applications, with documented use in cardiac and vascular diseases [182], nephrotic syndrome [183], and liver diseases [184]. Regarding oncogenicity, it is important to emphasize that the cells used by Heidmann et al. for transfection with retroviral TM envelope proteins were already tumorigenic in irradiated, immunosuppressed mice. Thus, TM protein expression represented a final but not essential step contributing to tumor formation in immunocompetent mice. Furthermore, tumor development appears to be relatively uncommon in pigs and has predominantly been reported in aged animals [185].

An alternative approach involves introducing vectors expressing p15E directly into the organ after its removal from the donor pig and prior to transplantation into the recipient, a strategy that has been well described in the literature [186]. In this proof-of-principle study of pig-to-pig heart transplantation, pig blood containing an adenoviral vector encoding a target gene was used to perfuse the donor pig heart, resulting in robust transgene overexpression. Following implantation of the treated heart, transplant survival was significantly prolonged [186]. This ex vivo delivery strategy offers the practical advantage of confining p15E expression to the transplanted organ itself, thereby avoiding the systemic immune concerns associated with whole-animal transduction. Applying this technology, the immunosuppressive TM protein encoded by HERVs could be exploited to attenuate rejection of allotransplants, offering a novel immunomodulatory avenue in allotransplantation.

## 15. Open Questions

The major unresolved questions concern the dual function of the isu domain in both viral replication and immunosuppression, as well as the identity of its still unknown receptor. The essential role of the isu domain during viral fusion and entry is evident. Deletion of a sequence encompassing the isu domain and the Cys–Cys loop of Mason–Pfizer monkey virus resulted in an Env precursor that was defective in transport [187]. Deletion of the highly conserved region of the isu domain (positions 1–11 in Figure 2) generated noninfectious particles due to disruption of the interaction between the SU and TM envelope proteins and subsequent shedding of gp70 into the culture medium [187].

In Friend murine leukemia virus (F-MuLV), mutation of two key residues within the isu domain (E14R and A20F) was reported to abolish immunosuppressive activity in the tumor rejection assay. In this model, viral replication competence is not required because the TM envelope protein is expressed on the surface of tumor cells, which are able to induce tumor formation in irradiated mice but not in immunocompetent mice [135]. Nevertheless, wild-type F-MuLV containing the intact isu domain replicated efficiently in vitro and in vivo in immunocompetent mice, whereas the virus carrying the mutated ISU domain replicated only in irradiated mice [135].

However, Kassiotis et al. demonstrated that these mutations impaired viral infectivity, but only when the virus was produced in cells expressing the receptor for the SU envelope protein [72]. They proposed that the isu domain provides structural stability to the SU envelope protein, enabling it to withstand the conformational changes induced upon interaction with the cellular receptor [72]. When they investigated the effect of synthetic isu peptides on T-cell responses, no immunosuppressive activity was detected [78]. This lack of activity may be explained by the use of extended monomeric peptides, as previous studies demonstrated that monomers are inactive, whereas peptides conjugated to BSA or peptide multimers exhibit immunosuppressive activity [50,65,77] (see Section 4).

Regardless of whether the ancient evolutionary origin and high conservation of the isu domain (see Section 2) are primarily related to its role in viral fusion and entry, this domain in intact virions, viral or recombinant TM envelope proteins, or peptide multimers is able to induce immunosuppression.

**Figure 7:**
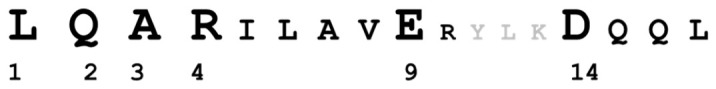
Schematic presentation of the influence of mutations in the isu domain of HIV-1 gp41 on IL-10 release; large letters indicate abrogation of IL-10 release (core region), small letters less inhibition, and amino acids given in gray (RYLK) were not investigated. Positions 1, 2, 3, 4, and 9 are also crucial for IL-6 release [106].

It is important to note that mutations affecting amino acids that are highly conserved across all retroviruses completely abolish the ability of recombinant gp41 produced in yeast to induce IL-10 and IL-6 production [106] (Figure 2 and Figure 7). These data suggest that the active isu domain may be discontinuous, with amino acids 1–4 constituting one functional region and amino acids 9–14 forming a second. Analysis of the isu domain polymorphisms of more than 2000 HIV-1 sequences did not reveal mutations of the type L1A, Q2A, A3G, R4A, L6A, A7G, E9A, and D14A in the isu domain of HIV-1 sequences from infected humans [106]. The isu sequence of human syncytin 1 (not immunosuppressive in the tumor rejection assay) and syncytin 2 (immunosuppressive) have differences in positions 6, 9, 13, 14, and 17 [133], which explains why only one is immunosuppressive. Murine syncytin-A (not immunosuppressive) and syncytin-B (immunosuppressive) differ mainly in the important position 14 [133].

The fact that the receptor for the isu domain remains unidentified, despite the description of numerous binding proteins using various approaches (see Section 8), represents a major obstacle. Although available data suggest that ISU-mediated signaling may involve the Ras-Raf-MEK-MAPK and PI3K-AKT-mTOR pathways, definitive evidence is still lacking. Based on current findings, the putative receptor appears to be broadly expressed across hematopoietic cell populations and is likely evolutionarily conserved. Further studies are required to identify this receptor and elucidate the underlying signaling mechanism.

Of particular interest is the question of why simian immunodeficiency viruses (SIV) are non-pathogenic in their natural hosts despite maintaining high viral loads (see Section 2). The observation that trans-species transmission of SIV from its natural host to humans or rhesus macaques results in AIDS suggests that the isu domain remains functionally active in the new host. The high viral burden in the natural host would be expected to result in substantial expression of the TM envelope protein, suggesting that natural hosts may have evolved mechanisms to counteract the immunosuppressive activity of this protein. Such mechanisms could involve disruption of TM envelope protein binding to its receptor or interference with downstream signal transduction pathways.

## 16. Conclusions

Overwhelming evidence indicates that the immunosuppressive (isu) domain within the TM envelope protein is a major contributor to the immunosuppressive properties of retroviruses. In infections with immunodeficiency viruses, which encode additional regulatory non-structural proteins, these proteins, such as Nef of HIV-1, can also contribute to immunosuppression. In certain cases, surface envelope proteins further enhance this effect. During the process of infection, viral envelope proteins, which are localized as surface-exposed knobs on the virus particle, are the first viral components to interact with the host immune system. Other viral proteins, such as Nef, are produced at later stages of infection.

The immunosuppressive TM envelope proteins of “enslaved” endogenous retroviruses, known as syncytins, play a crucial role in placentogenesis and in protecting the embryo from the maternal immune response. Similarly, in the tumor rejection model, which uses tumor cells able to induce tumors in irradiated but not immunocompetent mice, immunosuppressive retroviral TM proteins expressed on the surface of these cells significantly promote tumor progression. The same applies to naturally occurring tumors expressing p15E-related proteins.

Mutations within the isu domain appear to be a promising strategy for improving the efficacy of vaccines against retroviruses. Furthermore, if the expression of immunosuppressive TM proteins on the surface of xenotransplants can indeed reduce immune rejection, this could represent a major breakthrough in xenotransplantation.

## Figures and Tables

**Table 1:** Immunosuppressive activity of inactivated and purified/pelleted retrovirus preparations in vitro.

Virus Preparation	Inactivation, Purification	Inhibited Immune Reaction	References
Avian retrovirus, F-MuLV, MMTV	Pellet	Mitogen and antigen responses	Israel et al., 1979 [25], Wainberg et al., 1980 [26]
FeLV, MuLV	UV	Mitogen and antigen responses and MLR, suppress IL-2 release of murine splenocytes	Copelan et al., 1983 [27], Orosz et al., 1985 [28]
FeLV, RD-114	UV	Mixed lymphocyte reaction RD-114 not reactive	Stiff et al., 1983 [29]
FeLV	UV	Inhibition of erythroid colony-forming cells	Wellmann et al., 1984 [30]
FeLV	UV	Mitogen and antigen responses, suppress IL-2 release of murine splenocytes	Orosz et al., 1985 [31]
FeLV	UV	Suppression of in vitro neutrophil function	Lafrado et al., 1987 [32]
FeLV	UV	Inhibition of phorbol ester-induced neutrophil chemiluminescence	Dezzutti et al., 1990 [33]
R-MuLV	Freeze-thaw	Mitogen response and MLR	Fowler et al., 1977 [34]
Type D retrovirus PMFV, MPMV	Ether	Mitogen response of human PBMCs, MPMV not reactive	Denner et al., 1985 [23], Denner et al., 1985 [35]
BaEV, type D retrovirus PMFV, R-MuLV	Ether	Mitogen response of human PBMCs and murine spleen cells	Denner et al., 1980 [36], Denner et al., 1985 [23]
PERV	Ether, Triton X-100	Mitogen response of human PBMCs	Tacke et al., 2000 [37]
HTLV-1	UV	Mitogen response of human PBMCs	Wainberg et al., 1985 [38]
HERV-K, PERV	Pellet	IL-10 induction	Morozov et al., 2013 [39]
KoRV	Pellet	IL-10 induction	Fiebig et al., 2006 [40]
HIV-1	Triton X-100, UV, psoralene	T and B cells, bone marrow colonies	Hofman et al., 1989 [41]
HIV-1	UV	Mitogen, and allogen proliferation of T cells, decrease in IL-2	Amadori et al. 1988 [42]
HIV-1	AT-2	Antibody responses in human lymphoid tissue ex vivo	Fitzgerald et al., 2004 [43]

BaEV, baboon endogenous retrovirus; F-MuLV, Friend murine leukemia virus; FeLV, feline leukemia virus; RD-114, feline virus from a rhabdomyosarcoma cell line RD-114; MLR, mixed lymphocyte reaction; MMTV, mouse mamma tumorvirus; MPMV, Mason-Pfizer monkey virus; PERV, porcine endogenous retrovirus; PMFV, permanentes Mensch-Fibroblastenvirus (permanent human fibroblast virus); R-MuLV, Rauscher murine leukemia virus; HTLV-1, human T-lymphotropic virus; HERV-K, human endogenous retrovirus-K; KoRV, Koala retrovirus; HIV-1, human immunodeficiency virus-1; UV, ultraviolet; AT-2, aldrithiol-2.

**Table 2:** Immunosuppressive properties of recombinant (produced in bacteria or yeast) and viral TM envelope proteins including gp41 of HIV-1.

Retroviral TM Protein	Reaction	References
Purified p15E of FeLV	Inhibition of ConA-induced proliferation of normal feline lymphocytes, inhibition of capping of receptors for concanavalin A on normal feline lymphocytes, reduction of cytotoxic antibody titers in rats, support of tumor development in vivo	Mathes et al., 1979 [44], Copelan et al., 1983 [27]
Purified p15E of FeLV	Suppression of Con A receptor mobility	Nichols et al., 1979 [47]
Recombinant p15E endogenous MuLV	Down-regulating INFγ, up-regulating IL-10	Kim et al., 1999 [49]
Purified p15E of R-MuLV	Inhibition of interleukin-2-dependent proliferation of the murine T-cell line CTLL-2	Ruegg et al., 1989 [50]
Purified p15E of FeLV	Inhibition of mitogen and antigen responses	Orosz et al., 1985 [28]
Purified p15E of FeLV	Suppression of in vitro neutrophil function	Lafrado et al., 1987 [32]
Purified p15E of FeLV	Suppression of feline bone marrow fibroblast colony-forming units	Wellman et al., 1988 [51]
Purified p15E of FeLV	Inhibition of phorbol ester-induced neutrophil chemiluminescence	Dezzutti et al., 1990 [33]
Purified p15E of FeLV	Inhibition of erythroid colony-forming cells	Wellmann et al., 1984 [30]
Purified p20 of RD-114	Suppression of mitogen response of feline lymphocytes and Con A receptor capping	Olsen et al., 1981 [52]
Recombinant gp41 HIV	Production of IL-10 in monocytes, but not lymphocytes	Koutsonikoli et al., 1997 [53]
Recombinant gp41 HIV	Induction of IL-6 and IL-10 production in THP-1 cells	Takeshita et al., 1999 [54]
Recombinant gp41 HIV	Modulation of IL-10 and chemokine receptors on monocytes, astrocytes and neurones	Speth et al., 2000 [55]
Recombinant gp41 HIV	Induction of IL-10 in monocytes, but not in B, T, or NK cells, reduction of IL-2 and IFNγ	Barcova et al., 1998 [56]
Recombinant TM protein of HERV-K produced in yeast	Modulation of cytokine expression (cytokine array and microarray), modulation of the gene expression in human PBMCs, inhibition of mitogen response of human PBMCs and murine spleen cells, increase of IL-10 release	Morozov et al., 2013 [39]
Trimeric recombinant ectodomain of HIV-1 gp41 produced in human 293 cells	Modulation of cytokine expression (cytokine array), increase of IL-10 release, binding to monocytes and lymphocytes	Mühle et al., 2017 [57]
Recombinant syncytin-1	Inhibition of LPS/PHA-stimulated cytokine responses, inhibition of TNF-α, CXCL10 and IFN-γ	Tolosa et al., 2012 [58]

Con A, concanavalin A; gp41, glycoprotein 41,000 kDa; FeLV, feline leukemia virus; HERV-K, human endogenous retrovirus-K; HIV, human immunodeficiency virus; IL-6, interleukin-6; IL-10, interleukin-10; INF-γ, interferon γ; NK, natural killer; p15E, protein 15,000 kDa; R-MuLV, Rauscher murine leukemia virus; TM, transmembrane.

**Table 6:** Immunosuppressive properties of retroviral TM envelope proteins or isu domains in vivo.

Viral Protein, Peptide	Test System	Reference
FeLV	Vaccination study	Mathes et al., 1979 [44]
F-, Mo-, R-MuLV	Macrophage accumulation in mice	Cianciolo et al., 1980 [80]
Conjugates of CKS-17	Inhibition of delayed-type hypersensitivity to sheep erythrocytes in the feet of mice	Nelson et al., 1989 [86]
Mo-MuLV	Tumor rejection assay	Mangeney et al., 1998 [130]
MPMV	Tumor rejection assay	Blaise et al., 2001 [131]
HERV-H	Tumor rejection assay	Mangeney et al., 2001 [132]
human syncytin-2, mouse syncytin-B, ERV-3	Immunosuppressive, tumor rejection assay	Mangeney et al., 2007 [133]
human syncytin-1, mouse syncytin-A	Non-immunosuppressive, tumor rejection assay	Mangeney et al., 2007 [133]
F-MuLV	Virus infection, double mutation abolishes immunosuppression and infection	Schlecht-Louf et al., 2024 [134]

F-MuLV; Friend leukemia virus; Mo-MuLV, Moloney murine leukemia virus; R-MuLV, Rauscher murine leukemia virus; CKS-17, synthetic isu peptide of gammaretroviruses; MPMV, Mason-Pfizer monkey virus; HERV-H, human endogenous retrovirus H; ERV-3, endogenous retrovirus-3.

**Table 7:** Immunosuppressive properties of retroviral TM envelope proteins expressed on cell surfaces in vitro.

Virus	Test System	Reference
HIV-1 gp41	Ectodomain of gp41 expressed on target cells inhibited the antigen-specific response of murine CD8^+^ T cells by impairing their IFN-γ production.	Mühle et al., 2017 [57]
PERV virus or p15E	Expression of p15E induces expression and release of IL-10, IL-6 and others in human PBMCs, inhibition of cytotoxic cells, down-regulation of MHC-I	Denner et al., 2026 [113]

HIV-1, human immunodeficiency virus-1; PERV, porcine endogenous retrovirus.

**Table 8:** Sequence homology between CKS-17 (the isu domain of MuLV, FeLV, KoRV, GaLV, and PERV) and the isu sequence in HERV-H [174]. Identical amino acids are marked red.

Protein	Sequence (Amino Acids)																
CKS-17	** L **	** Q **	** N **	** R **	** R **	** G **	** L **	** D **	** L **	** L **	**F**	**L**	**K**	**E**	** G **	** G **	** L **
HERV-H 19, 10	** L **	** Q **	** N **	** R **	** R **	** G **	** L **	** D **	** L **	** L **	**T**	**A**	**E**	**K**	** G **	** G **	** L **
HERV-H 18	** L **	** Q **	** N **	** R **	** R **	** G **	** L **	** D **	** L **	** L **	**N**	**A**	**E**	**K**	** G **	** G **	** L **

## Data Availability

No new data were created or analyzed in this study. Data sharing is not applicable to this article.
